# Malarial Hæmoglobinuria

**Published:** 1891-08

**Authors:** E. H. M. Parham

**Affiliations:** Fordyce, Ark.


### Southern Medical Record.

A MONHLY JOURNAL OF MEDICINE AND SURGERY.

VOL. XXI. ATLANTA, GA., AUGUST, 1891. No. 8

### Original Articles.

### MALARIAL HÆMOGLOBINURIA.

BY E. H. M. PARHAM, M. D., FORDYCE, ARK.

The above disease, is one that has attracted a great deal of attention from the medical profession, and its etiology, pathology and treatment freely discussed in the medical literature of the country, by men of the first rank of the medical profession, and it is astonishing to see how conflicting is the treatment proposed by different writers.

I have before me an article, in pamphlet form, written by Dr. McHatton, of Macon, Ga., and reviewing the treatment he proposes, I feel constrained to exclaim, in the language of a distinguished and ancient orator, "Quo usque tandem abutere, patientia nostra." I find no objection to his etiology of this disease, as being unquestionably malarial, neither do I find his symptomatolgy defective, and certainly he deserves a great deal of credit for his numerous and critical analyses of the urine taken from those afflicted with this disease, and has thrown some light on the true state of the blood and morbid secretions of the kidneys, and after his lengthy research and thorough investigation, he seems to have arrived at the sad conclusion that quinine is the only reliable remedy we have, calculated to exercise salutary influences over that malady; without stating what power quinine has, or its mode of action, either in controlling hemorrhage or correcting morbid secretion, or favoring the elimination of effete substances contained in the blood, all of which I view, as of vital importance. I have always considered quinine (I mean the effects of it) as predisposing to hemorrhage and therefore should not be used when the hemorrhage already exists, and I do not know that the power of correcting morbid secretion has ever been claimed for it, and I think that both of the above indications are important and should be promptly met.

I speak from clinical experience alone, when I say that hæmoglobinuria ceases to be considered a formidable disease in South Arkansas since what we call the quinine treatment has been abandoned, and I am sure that there are very few reputable physicians, if any, who have any considerable experience in treatment of this disease, and in the confines of my acquaintance, who would think of prescribing quinine.

Malarial hematuria, or hæmoglobinuria, as it occurs in this part of our common country, (I never care to go to foreign countries to seek statistics, when we have a sufficient number of cases at home) is always a continued fever and after the initial paroxysm is never attended with other rigors, except when quinine in sufficient quantity to produce a shock on the nerve centres is used, the severity of which shocks, and the aggravation of other symptoms is always in proportion to the amount of quinine used—for the confirmation of which fact, I respectfully refer to the clinical experience of Drs. Hodge and Lea, of Princeton, Ark., March, and Williams, of Fordyce, Ark., and a host of other experienced physicians, if necessary. I speak of malarial hematuria, as it appears in South Arkansas, and not as it appears in foreign countries. I agree with Dr. McHatton, that chemical analysis is of great utility; but it will do no good unless it leads to a successful course of treatment.

I need not repeat here the symptoms of the disease under consideration, as Dr. McHatton has fully and correctly described them in his article.

TREATMENT.—I control the hemorrhage and alter the abnormal condition of the blood with the following:

℞ Ergotole,
Tinct. Iron, a. a. gtt. x.
Mix. Sig.: One dose, which should be repeated every four hours. It is best to use buchu-leaf tea, as the vehicle for the administration of the above, as it assists in removing effete substances from the blood, as well as it is calculated to restore the kidneys to their normal action. Quinine has no powers calculated to meet the above indications, and yet I view them as important, as I have often seen the hemorrhage truly frightful.

To quiet the sick stomach, correct morbid secretion of the liver and promote diaphoresis, I use the following:

℞. Calomel, gr. x.
Dover's Powder, gr. xxv.

Mix, and divide into 10 powders. Sig.: One every 4 hours, to be given alternately with the first prescription. I cannot see any reason to believe that quinine has any tendency to meet the above indications, and yet it cannot be denied, but that they are important. I have often obtained decided benefit from the application of a large blister over the liver, stomach and spleen, and never any unpleasant consequences. The bowels should be regulated by means of an emetic. (?)

The diet, of course, should be easily digested and nourishing.

Tinct. Iron, Tinct. Nux Vomica and Phosphoric acid, in proper proportions and doses, make a good tonic to be used before meals during convalescence.

I have written the above, hoping that it may induce some members of our profession to give it a trial and thereby learn that quinine is not a specific for all malarial troubles, and that although a wonderful remedy, may be abused as well as others.

With all due respect for the opinion of Dr. McHatton, I offer this for publication, having alone the good of the human family in view, knowing that on account of my age, I will be forced to retire from the practice of our profession before a great many years.

I will here state in conclusion, that the above treatment, in my hands, has been pre-eminently satisfactory up to the present time.

